# Health system reform in peri-urban communities: an exploratory study of policy strategies towards healthcare worker reform in Epworth, Zimbabwe

**DOI:** 10.3402/gha.v9.32219

**Published:** 2016-12-09

**Authors:** Bernard Hope Taderera, Stephen James Heinrich Hendricks, Yogan Pillay

**Affiliations:** 1 School of Health Systems and Public Health, University of Pretoria, Pretoria, South Africa; 2 National Department of Health of the Republic of South Africa, Pretoria, South Africa

**Keywords:** health, worker, reform, strategies, peri-urban

## Abstract

**Background:**

Human resources for health (HRH) remains a critical challenge, according to the Kampala Declaration and Agenda for Global Action of 2008 and the 2030 Sustainable Development Agenda. Available literature on health system reforms does not provide a detailed narrative on strategies that have been used to reform HRH challenges in peri-urban communities. This study explores such strategies implemented in Epworth, Zimbabwe, during 2009–2014, and the implications these strategies might have on other peri-urban areas.

**Design:**

Qualitative and quantitative methods were used in an exploratory and cross-sectional design. Purposive sampling was used to select key informants, a sample of healthcare workers that participated in in-depth interviews and community members who took part in focus group discussions. Secondary data were collected through a documentary search. Qualitative data were analysed through thematic analysis. Quantitative secondary data were examined using descriptive statistics and then compared with qualitative data to reinforce analysis.

**Results:**

The HRH reform policy strategies that were identified included ministerial intervention; policy review; and revival of the human resource for health planning, financial planning, multi-sector collaboration, and community engagement. These had some positive effects; however, desired outcomes were undermined by financial, material, human resource, and social constraints.

**Conclusions:**

Despite constraints, the strategies helped revive the health delivery system in Epworth. In turn, this had a favourable outlook on post-2008 efforts by the Global Health Alliance towards healthcare worker reform and the 2030 Sustainable Development Agenda in peri-urban communities.

## Introduction

Whilst a large body of knowledge has been accumulated on strategies towards health system reforms, there is no detailed description on healthcare worker reform strategies in peri-urban communities (1, 2). This has often resulted in a misalignment between human resources for health (HRH) and the health systems reform process and contributed to serious healthcare worker challenges worldwide (3). In answer to these challenges, the Kampala Declaration in 2008 (the result of which was the Global Forum on HRH) called for a global commitment to combat the critical shortages of health workers (4, 5). This commitment has subsequently been reinforced through the 2030 Sustainable Development Goals 3 and 11, which indicated that healthcare worker reorganisation in peri-urban areas is critical towards providing universal health coverage (6).

Peri-urban areas (those located between urban and rural) have emerged as borderline communities of recent times, typified by an expansion of disorganised informal settlements (7). In a bid to integrate these areas with other communities, the Government of Zimbabwe has incorporated them into the local governance system by regularising them as local boards. Local boards are a type of municipality that occupy the lowest position in the hierarchy of the local municipal authorities’ structure in Zimbabwe. Local boards normally require state assistance towards regularisation and policy implementation. In Zimbabwe, there are four local boards, which have been regularised, of which Epworth is one (8, 9). Before 2009, the health system reform in Epworth was severely undermined by an unfavourable prevailing macro-economic situation. These challenges had a serious negative effect on the healthcare worker system, undermining the local health system reform process (10). Between 2009 and 2014, healthcare worker reform strategies were pursued in this peri-urban community (11, 12). This study explores healthcare worker reform strategies between 2009 and 2014 in Epworth, and the implications that these strategies have had and whether results could be applicable in other peri-urban areas in the context of the post-2008 policy agenda.

## Subjects and methods

### Study setting

**Fig. 1:**
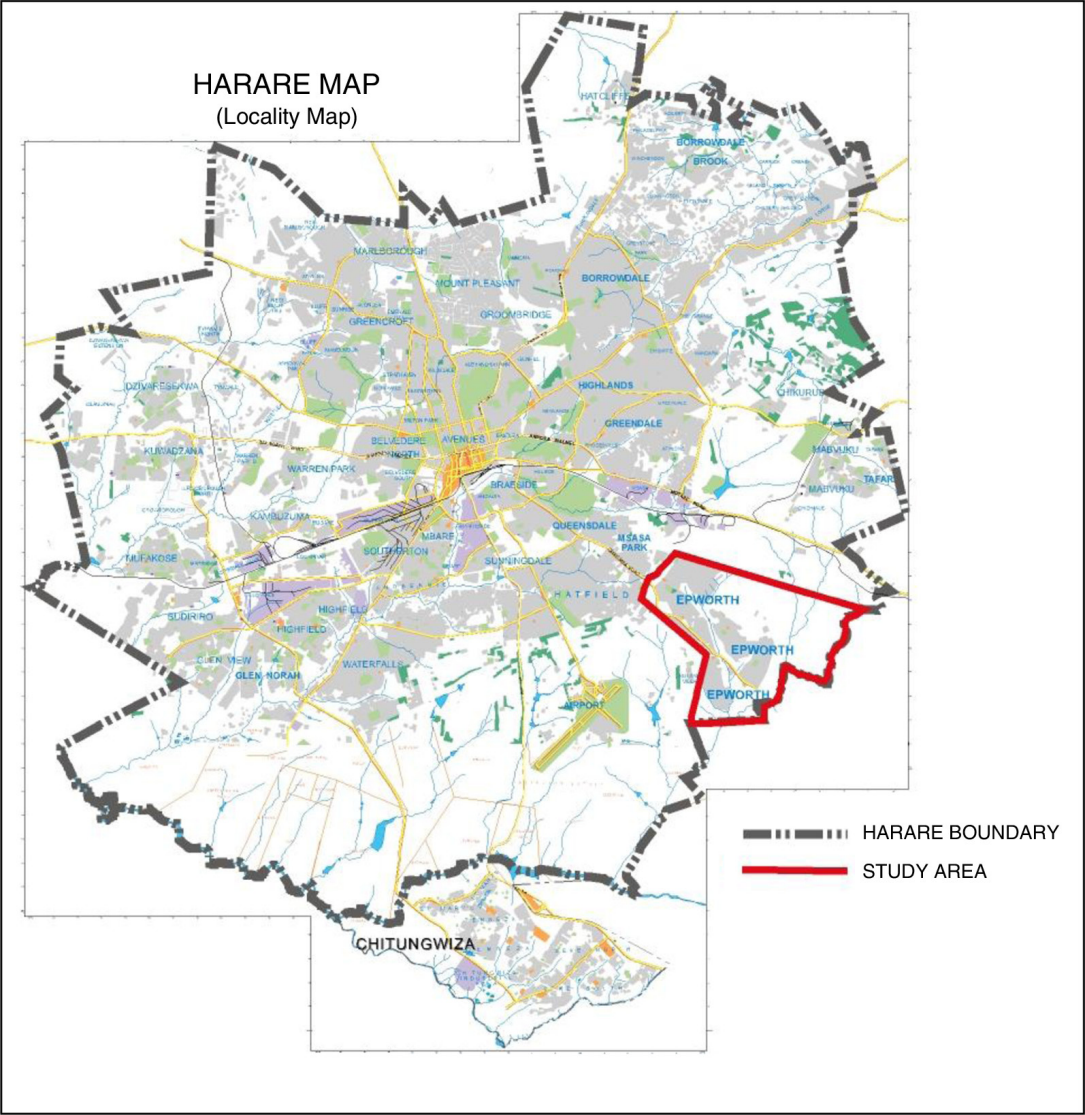
The location of Epworth on the Harare map.(Reproduced with permission from Ref. (9).)

This study was carried out in Epworth, a peri-urban area in south-east Harare (Fig. 1).

Epworth comprises seven wards containing 10 local clinics: two local board clinics, one mission clinic, and seven private clinics. Between 2009 and 2014, healthcare worker reform strategies were implemented through these local health institutions and the community. Data were collected in this area and in Harare from July to December 2015.

### Study design

A cross-sectional design was used for an exploratory qualitative study in which primary data were collected from multiple sources that included key informant interviews, in-depth interviews, focus group discussions (FGDs), and non-participant observation (13, 14). Interviews were open-ended and semi-structured following a guide that covered topics of HRH reform interventions, community engagement, and service delivery outcomes. Secondary sources included quantitative data collected through a documentary search. Multiple data sources were used to enable the generation of a comprehensive and valid data set. Qualitative and quantitative data sets were then combined to cross-verify and complement each other in the presentation and analysis of findings (14).

### Study participants, sampling, and data collection procedures

#### Qualitative study

The primary and secondary data were collected by the first author at principal and agent levels as outlined in Table 1. To start with, a documentary search was carried out to determine the reform policy content, interventions, and outcomes with regard to human resource for health reform in Zimbabwe and Epworth between 2009 and 2014. From this, a key informant interview guide was developed and piloted through two key informant interviews with policy makers from the Ministry of Health (MoH). After this, the interview guide was used in five key informant interviews with purposively selected policy makers drawn from the MoH, Health Services Board (HSB), and the Zimbabwe Association of Church Hospitals (ZACH) national offices in Harare and the Mashonaland East Provincial Medical Office (MEPMO) in Marondera, Zimbabwe. Other equipment used included a digital audio recorder, notebooks, and pens. Data collected from primary and secondary sources at the principal level were then used to develop an interview guide for the local-level key informant interviews, in-depth interviews, and sample interview guides.

**Table 1:** The setting, level, number, and type of study participants interviewed

Setting	Level	Type of participants	Type of interviews	Number of interviews
MoH	Principal	Policy makers	Key informant	3
HSB	Principal	Policy makers	Key informant	2
ZACH	Principal	Policy makers	Key informant	1
MEPMO	Principal	Policy makers	Key informant	1
ELB/local clinics	Local/agent	Policy makers	Key informant	3
Local clinics	Local/agent	Sisters in charge/personnel managers	In-depth	7
Local clinics	Local/agent	Healthcare workers	Sample	19
Community	Local/agent	Community members	Focus group discussions (10 people each)	5

Source: Fieldwork data set.

From this, three key informant interviews were carried out by the first author with purposively selected local-level policy makers drawn from the Epworth Local Board. Data were collected to determine policy adoption, context, community-level interventions, and outcomes. Interview guides for key informant and in-depth interviews were piloted during the first interview for each category at this local level. In total, seven in-depth interviews were carried out with purposively selected sisters in charge/personnel managers at local health clinics. Through these in-depth interviews, we explored clinic-level reform strategies and compared outcomes of the 2009–2014 reform interventions with the situation before this period depending on the availability of data sources, which enabled us to trace data collected way back in 2007. Data from these in-depth interviews were then used to refine interview guides used for 19 sample interviews with purposively selected healthcare workers drawn from local clinics. The first three of these interviews were used to pilot the interview guide. Selection was based on the availability and willingness of healthcare workers to take part in the qualitative study. Each interview lasted between 30 and 45 min. In this, we explored outcomes of the HRH reform policy interventions on healthcare workers. Community members were recruited into five FGDs of 10 people each to determine community participation, engagement strategies, and service delivery outcomes. Of these, two were carried out with purposively selected community health workers/peer educators. Other equipment used included audio digital recorders, pens, and notebooks. Data were collected from each category until saturation was reached. This also determined the number of interviews carried out in each category.

#### Quantitative study

Quantitative secondary data were collected through a documentary search at local clinics to explore numerical outcomes on aspects that included staffing levels, sector contributions towards HRH, and doctor-to-patient ratios. Priority was placed on staffing levels because they are a critical issue in the current global focus.

### Ethical clearance

The study was part of a wider PhD in Public Health research project, to develop an application towards determining how national HRH policy interventions impact local HRH systems in peri-urban communities using Decision Space Mapping Analysis. The protocol received approval from the Academic Advisory Committee, the Research Ethics Committee of the University of Pretoria, South Africa (Reference number 413/2014), and the Medical Research Council of Zimbabwe (Approval Number MRCZ/A/1941). Written informed consent was obtained from all participants, who were approached and took part in the research.

### Presentation and discussion of findings

Patterns were identified across transcribed qualitative data sets, which were presented narratively. The patterns were then coded and grouped manually by the main author. Discussions amongst all authors resulted in the emergence of five themes in which data were then subjected to analysis. Thematic analysis suited the large data set and allowed the categorisation of data for easier interpretation using NVivo (14). Quantitative secondary data on staffing levels were tabulated and examined using descriptive statistics. Qualitative and quantitative data sets were then integrated during presentation and analysis to facilitate cross-verification and more comprehensive interpretation of aspects that included staffing levels, sector contributions towards HRH, and doctor-to-patient ratios (13, 14).

## Results

### Ministerial intervention

We established from the MoH, HSB, and ZACH that the health ministry intervened in Epworth by setting an HRH Taskforce that had structures at national, provincial, and district levels, through which reform interventions were implemented. Each taskforce was made up of representatives from line ministries and the health civil society, predominantly made up of donor organisations from the Zimbabwe United Nations Development Assistance Framework. For Epworth, ministerial intervention was undertaken through the Provincial Medical Office Health Executive of Mashonaland East that also consisted of the Seke District Medical Office that represented the Seke district health executive and the Seke district health management team under which Epworth fell. Through these structures, the taskforce facilitated the implementation of healthcare worker reform policy interventions between 2009 and 2014. We established from local key informants that intervention by the principal was inevitable, necessitated by capacity constraints that emanated from a narrow revenue base, semi-formal settlement, immigration, impoverishment, and an overwhelming disease burden for the Epworth Local Board.

### Policy review

The Global Political Agreement of 2008 marked a watershed point at which the MoH reviewed the health reform policy at the national level. This review was guided by the World Health Organization Country Cooperation Strategy and resulted in the formulation of the National Health Strategy of 2009–2013. A documentary search revealed that one of the policy instruments of this National Health Strategy was the HRH Policy and Strategy. In line with this HRH Policy and Strategy, the HRH Policy of 2009–2014 was formulated and implemented through the HRH Strategic Plan of 2010 and 2014. This HRH policy consisted of four main result areas that included HRH planning and financing; production, training, and development; deployment, utilisation, and management; and Human Resource Information and Research (11, 12). This strategic-level policy framework laid a foundation upon which HRH reform was pursued in Epworth from 2009 to 2014.

### Revival of the healthcare worker planning process

According to local key informants and the HSB, human resource planning was revived as part of the reform process. For the two local board clinics and the mission clinic, the MoH intervened through regular inquiries through district and provincial medical offices, and engagement with the local board. Findings suggested that this impacted positively on the local healthcare worker system. To start, human resource planning, which had literally ceased in the midst of the socio-economic challenges of pre-2009, became functional again. This contributed towards reducing the gap between the demand and supply of healthcare workers between 2009 and 2014. However, the arrangement was different for local private clinics as they were guided by their smaller financial capacity in human resource planning; they contributed with only 11 of the 56 nurses and 6 of the 27 nurse aides, as outlined in Table 2.

**Table 2:** Sector contributions towards human resources for health

	Public sector	Private sector
Nurses	45	11
Nurse aides	21	6
Totals	76	17

Source: Fieldwork data set.

We observed that the private sector contribution did not do much to help overcome the shortage of nurses that persisted in this community by the end of 2014. These shortages were manifested by a heavier workload for healthcare workers at the two municipal clinics and one mission clinic compared with the private clinics where there were also barriers to access that included higher service costs with no option for free medical treatment and fixed consultation times.

### Complementary effort towards financial planning

From the key informants at the local board, MoH, HSB, and ZACH, we determined that the financial planning between 2009 and 2014 was characterised by parallel and complementary human resource budget structures. The MoH incorporated the three public clinics in Epworth into national funding structures to help finance salaries of healthcare workers from its share of the national budget. To counter the effect of high inflation of pre-2008, a multicurrency regime in which all salaries were paid in US dollars was adopted. We established from healthcare workers that this intervention had a positive impact, as it helped bring stability to their salaries. However, most healthcare workers stated that their salaries were not adequate enough to meet the basic needs, including transport, food, and clothing required for a whole month, which left them demoralised and concerned (Table 3) (9).

To help cope with this situation, the Epworth Local Board and Mission used their local budgets to pay top-up allowances during the middle of each month. We established that this strategy impacted positively on healthcare workers, as it helped supplement their salaries that would have run out by that time. However, incapacity to pay this top-up allowance to all healthcare workers at the three local public clinics undermined the realisation of desired outcomes. For instance, only four of the eight nurses and three of the four nurse aides that had been working at the mission clinic before 2009 received the salary top-up allowance. The rest of the staff that also included two primary counsellors and one environmental health officer never benefited from this intervention. This scenario created a feeling of exclusion and division amongst the healthcare workers (9). The MoH was also incapacitated to intervene in this, because the budgetary allocation towards health from the share of the national budget also fell short of the 15% minimum requirement set out in the 2000 Abuja Declaration which suggested financial constraints (15, 16). Other competing macro-economic priorities that included the staff-monitored programme by the government and the international monetary fund restricted expenditure by the MoH in Epworth (17).

### Multi-sector collaboration to expand the human resource base

Collaboration consisted of the MoH, local board, mission, private sector, community members, and an international NGO. We established from key informants drawn from the MoH, HSB, and the local board that this helped address the critical shortage of healthcare workers that existed before 2009 when there were only two clinics: the mission clinic and a local board clinic. Data from a documentary search indicated that in 2007, the mission only had four nurses and three nurse aides. The nurse-to-patient ratio was 35:10,000 compared with 13:10,000 for the rest of Zimbabwe in 2011 (18, 19).

**Table 3:** Numbers of healthcare workers in Epworth before 2009

Type of clinic	Nurses	Non-medical healthcare workers
Local board clinic	10 nurses 3 midwives	2 nurse aides 1 environmental health officer 1 dispensary assistant
Mission clinic	4 nurses	3 nurse aides
Total	17	7

Source: Fieldwork data set.

In addition, there were only 10 nurses, three midwives, one environmental health officer, and two nurse aides at the local board clinic. These were inadequate to meet the health needs of an average population of about 114,000 people at that time. The MoH engaged an international NGO, through an HIV/AIDS- and TB programme-specific partnership and healthcare worker reorganisation. This helped contribute towards the improved numbers of healthcare workers, as outlined in Table 4.

**Table 4:** Increased number of healthcare workers at clinics in Epworth by 2014

Type of clinic	Personnel managers	Nurses	Non-medical healthcare workers	Total for all cadres^a^
Mission clinic	1 sister in charge	2 primary counsellors; 6 nurses; 2 primary care nurses	1 environmental health officer/technician; 4 nurse aides	15
Local board clinic 1	1 sister in charge	11 nurses; 6 midwives; 1 state-certified nurse; 3 primary care nurses; 2 primary counsellors	1 pharmacy technician; 3 laboratory scientists; 3 ambulance drivers; 1 environmental health officer; 11 nurse aides	42
Local board clinic 2	1 sister in charge	13 nurses	1 dispensary assistant; 1 environmental technician; 5 nurse aides; 1 pharmacy technician	21
Local private clinic	1 medical doctor	1 nurse; 1 primary care nurse	2 nurse aides	4
Local private clinic	1 medical doctor	3 nurses	4 nurse aides	7
Local private clinic	1 medical doctor	1 nurse; 1 midwife	2 nurse aides	4
Local private clinic	1 medical doctor	2 nurses	1 nurse aide	3
Local private clinic	1 medical doctor	1 primary care nurse	0	1
Local private clinic	1 medical doctor	2 nurses	0	2
Local private clinic	1 medical doctor		2 nurse aides	2
Total		56	45	101

a The total of all cadres does not include medical doctors, sisters in charge, and medical doctors from the international NGO (Source: Fieldwork data set).

However, from the FGDs with community members, it was noted that private medical doctors provided services at fixed times of the day and required a consultation fee, which made their services out of reach to most members of the community. As a result, we established that most locals had no choice but to access the three local public sector clinics. This impacted negatively on healthcare workers at these clinics. To help cope with this scenario, we established from local key informants that there was community engagement by the local board, health ministry, and international NGOs. This resulted in the revival of the Community/Village Healthcare Worker Programme and initiation of the Peer Educators Programme. From this, a pool of about 30 community health volunteers was deployed into the community wards. Community/village health workers complemented on-site medical health personnel in performing outreach work, whilst peer educators were HIV-positive volunteers deployed on-site to provide assistance in the non-medical aspects of service delivery (9). However, there were financial and material constraints that undermined their work. The community/village health workers revealed these constraints by stating that:

our ability to reach out into more parts of the community are undermined by the fact that we are short-staffed. You find that there will be an average of two or three of us working in a ward, against the initial standard of five. As a result, this makes it difficult for us to reach out into a ward of well over 1,000 households which makes the work overwhelming. Also we lose colleagues due to relocation to other jobs, which makes it more difficult for us. We are also not happy with unfulfilled promises which include bicycles, first aid kit, the need to recruit more CHWs to bring the numbers back to five per ward, and monthly allowances which we have not been paid by the Ministry of Health for 11 months now. (Community Health Workers; 7)

In addition, there were also social challenges that impacted negatively on peer educators who stated the following:

There is need for us to be provided with protective clothing to protect us from contracting diseases whilst working at the clinic. In addition, providing us with uniforms will make us appear equal in the eyes of the patients because some of us are very poor to the point that our clothes expose our poverty which is discomforting when working. At least uniforms will help conceal our poverty and make us more accepted and treated with respected by patients. In addition, the unavailability of uniforms makes it easier for us to be recognised as being HIV positive which exposes us to stigma. As a result, some patients call us names in such a way which is dehumanising and you do not feel good volunteering to provide your services each time you are ill-treated like that. We are also human and we deserve to be treated with dignity and respect like everyone else. (Peer Educator; 3)

Regardless of these constraints, community members stated that the deployment of more healthcare workers helped improve service delivery and health outcomes. There were, however, still concerns amongst community members over the staff shortages revealed through long queues, the negative attitude of healthcare workers towards patients, and the unavailability of some medical products.

## Discussion

The first theme identified in this study was ministerial intervention. Through its national, provincial, and district medical structures, the MoH intervened through HRH taskforces to provide technical, financial, and human resource assistance to the healthcare worker reform process in Epworth peri-urban area between 2009 and 2014. This helped to overcome the lack of capacity by the local board, which would have undermined the process. State intervention in local health systems is an idea in line with the neo-conservative strand of the new right perspective postulated by Bradshaw and North. In that context, direct state intervention, or through philanthropy, provides resources to facilitate health policy and practice (20). In this regard, our results suggested that intervention by MoH meant resources that enabled the revival of the healthcare worker reform in Epworth.

Policy appraisal was identified as our second theme. This is because our results suggested that the formulation of the 2009 NHS that set forth the HRH Policy and Strategy, the result of which was the 2009 to 2014 HRH Policy (11, 12). It has also been acknowledged in the literature on HRH reform that health policies are crucial tools that help facilitate planning, support decision-making, provide a framework for evaluating performance, help rally professionals and other sectors around problems, and legitimise reform actions (21). In Epworth, the healthcare worker reform policy framework that was formulated provided a strategic direction towards which all reform interventions of 2009–2014 were pursued.

**Fig. 2:**
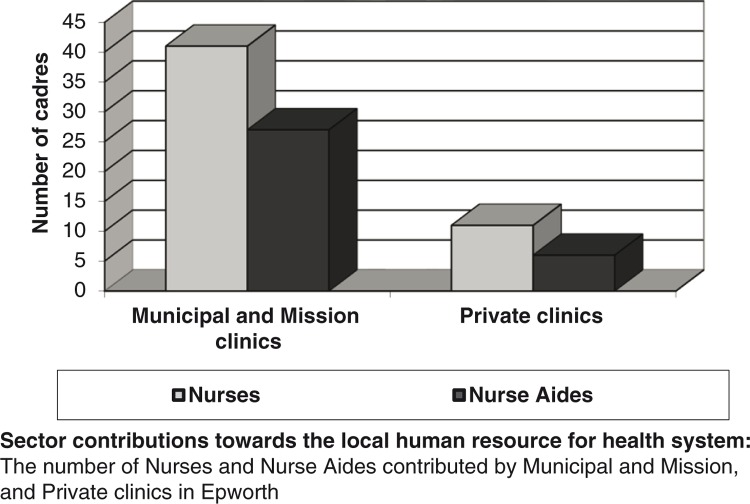
Sector contributions towards the local human resource for health system.Source: Fieldwork data set.

In this regard, we identified HRH planning as our third theme. The MoH engaged the local board through its provincial and district medical structures in human resource planning. Our results suggest that this impacted positively on the community as HRH planning became functional again, towards narrowing the gap between demand and supply of healthcare workers. However, we noted that the arrangement was different for local private clinics, and it seemed that this accounted for their smaller contribution of 11 out of the 56 nurses and 6 out of the 27 nurse aides (Fig. 2).

Apart from this, financial planning was identified as the fourth theme. That was based on our findings that suggested that financial planning between 2009 and 2014 was characterised by a collaborative engagement between the MoH, Epworth Local Board, and the mission. This facilitated the payment of salaries from the civil service wage bill by the MoH. It also enabled the local board and the mission to help supplement government effort through top-up allowances paid to healthcare workers at three local public sector clinics. In Malawi, the salary top-up scheme helped improve the working conditions of civil service healthcare personnel (22). However, whilst intervention at the three local public sector clinics had a positive impact on healthcare workers, it was undermined by the failure to reach out to all health personnel (9). Our findings also further suggested that the MoH was financially constrained to intervene on this initiative, which also compounded the situation.

**Fig. 3:**
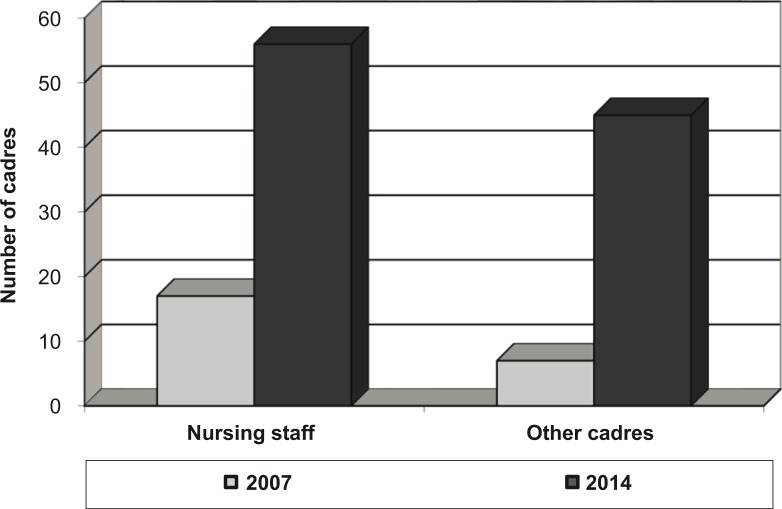
Increase in the number of nurses and other cadres between 2007 and 2014.Source: Fieldwork data set.

Multi-sector collaboration involving the MoH, international NGO, local board, mission, private sector, and community members was identified as our fifth theme. This resulted in an increase in the total number of nursing staff by 229% from 17 before 2009 to 56 in 2014. In addition, the number of other cadres increased by 543% from 7 in 2007 to 45 in 2014, as outlined in the Fig. 3.

It is worth noting that the percentage increase in the number of health cadres was greater than the population increase between 2002 and 2012. In 2002, Epworth's average population of 141,067 grew by 15% to 161,840 by 2012. This implies that the increase in the number of health personnel was significant and notable.

In addition, the physician-to-patient ratio of 8:100,000 was favourable when compared with that in the rest of the country in 2009, which was 6:100,000, and 3:100,000 in Tanzania (2012), 4:100,000 in Mozambique (2012), and 2:100,000 in Malawi (2009) (18, 19). However, this improvement was undermined by factors that include the unavailability of permanent physicians at the three local public sector clinics, reliance on programme-specific physicians from the international NGO, and cost and service time barriers associated with local private-sector physicians. Compounding this was a nurse-to-patient ratio that compared less favourably with that of the rest of the country in 2011, which meant the shortage of nurses had not been resolved by the end of 2014. This resulted in a heavier workload, in a high-pressure and high-risk work environment that characterised the three local public-sector clinics. The deployment outcome in Epworth was also less favourable against the WHO view that fewer than 23 medical health workers (physicians, nurses, and midwives) per 10,000 people would be insufficient to achieve coverage of primary healthcare needs. However, this position also appeared unattainable for other countries in the region, which attained better ratios during that period (19). For Epworth, this was further compounded by inadequate human, financial, and material resources, and stigma against peer educators that undermined the benefit of community engagement through the Community Health Volunteers Initiative.

## Conclusions

Healthcare worker reform strategies that were implemented in Epworth peri-urban community from 2009 to 2014 helped transform the local health delivery system. The strategies implemented towards this end included ministerial intervention, which helped overcome the lack of technical, financial, and human resource capacity by the Epworth Local Board, without which it might have been difficult, if not impossible, to initiate healthcare worker reform from 2009 to 2014. The policy review by the MoH that resulted in the formulation of the 2009–2014 HRH policy was also critical, as it provided strategic direction towards which HRH reform was pursued. The policy review contributed towards reform interventions that included revival of the HRH planning process, financial planning, and the establishment of a local multi-sector collaboration. These interventions had a favourable impact on the local HRH system compared with the situation before 2009. The current reform effort must however be sustained and reinforced towards better outcomes in deployment levels, salaries, and allowances; local financial planning; community engagement in health; and multi-sector collaboration to cope with the desired outcomes set by the Global Health Workforce Alliance 2008 and the Sustainable Development Agenda of 2030.

## Paper context

The need for healthcare worker reform is a critical challenge undermining the health system reform agenda in peri-urban communities. This study shows that policy strategies implemented in Epworth peri-urban community between 2009 and 2014 helped revive healthcare worker reform. These strategies included ministerial intervention, policy review, human resource and financial planning, multi-sector collaboration, and community engagement. There is need to reinforce and sustain healthcare worker reform effort in peri-urban communities towards providing universal health coverage.
